# MicroRNAs or Long Noncoding RNAs in Diagnosis and Prognosis of Coronary Artery Disease

**DOI:** 10.14336/AD.2018.0617

**Published:** 2019-04-01

**Authors:** Yuan Zhang, Lei Zhang, Yu Wang, Han Ding, Sheng Xue, Hongzhao Qi, Peifeng Li

**Affiliations:** Institute for Translational Medicine, Qingdao University, Deng Zhou Road 38, Qingdao 266021, China

**Keywords:** miRNAs, lncRNAs, coronary artery disease, atherosclerotic plaque

## Abstract

Coronary artery disease (CAD) is the result of atherosclerotic plaque development in the wall of the coronary arteries. The underlying mechanism involves atherosclerosis of the arteries of the heart which is a relatively complex process comprising several steps. In CAD, atherosclerosis induces functional and structural changes. The pathogenesis of CAD results from various changes in and interactions between multiple cell types in the artery walls; these changes mainly include endothelial cell (EC) dysfunction, vascular smooth muscle cell (SMC) alteration, lipid deposition and macrophage activation. Various blood markers associated with an increased risk for cardiovascular endpoints have been identified; however, few have yet been shown to have a diagnostic impact or important clinical implications that would affect patient management. Noncoding RNAs, especially microRNAs (miRNAs) and long noncoding RNAs (lncRNAs), can be stable in plasma and other body fluids and could therefore serve as biomarkers for some diseases. Many studies have shown that some miRNAs and lncRNAs play key roles in heart and vascular development and in cardiac pathophysiology. Thus, we summarize here the latest research progress, focusing on the molecular mechanism of miRNAs and lncRNAs in CAD, with the intent of seeking new targets for the treatment of heart disease.

Cardiovascular diseases (CVDs), especially coronary artery disease (CAD), remain the major cause of death worldwide and cause a major socioeconomic burden.

CAD results from atherosclerotic plaque development in the wall of the coronary arteries when the arteries that supply blood to the heart muscle become hardened and narrowed [1, 2]. Atherosclerosis is a progressive inflammatory disease and is the main underlying mechanism of CAD. Endothelial cell (EC) dysfunction caused by oxidative, hemodynamic, or biochemical stimuli initiates the development of atherosclerosis [3]. In turn, the permeability of ECs changes, and macrophages accumulate and release inflammatory mediators. Smooth muscle cells (SMCs) are then activated and begin proliferating and migrating [4, 5]. Therefore, the pathogenesis of CAD results from numerous changes in and interactions between multiple cell types in the artery walls; these changes mainly include lipid deposition, EC dysfunction, macrophage activation, and SMC alteration.

Various blood markers associated with an increased risk for cardiovascular endpoints have been identified; however, few have yet been shown to have a diagnostic impact or important clinical implication that would affect patient management [6]. Therefore, there is a great interest in innovative biomarkers that can be used to assess the risk of atherosclerosis and CAD progression as well as the efficacy of therapy [7, 8].

Many studies have shown that the occurrence and development of cardiovascular diseases are closely related with regulatory noncoding RNAs (ncRNAs), which include microRNA (miRNA), short interfering RNAs (siRNAs), and long noncoding RNAs (lncRNAs). Recent research on miRNAs and lncRNAs in heart disease has progressed rapidly [9]. The expression signatures of miRNAs and lncRNAs in tissues and blood have a potential role in the diagnosis and prognosis of disease and in the assessment of therapy. In the cardiovascular system, ncRNAs not only are vital for heart and vascular development but also play an essential role in the pathophysiology of cardiac abnormalities such as CAD [10, 11]. In addition, miRNAs and lncRNAs can be stable in plasma and other body fluids and could therefore serve as biomarkers for some diseases [12]. Thus, we summarize here the latest research progress, focusing on the molecular mechanism of miRNAs and lncRNAs in CAD, in an aim to seek new targets for the treatment of heart disease.

## The biogenesis and functions of miRNAs and lncRNAs

Noncoding RNAs are a class of genetic, epigenetic and translational regulators that contain short and long transcripts and have intriguing abilities for use as biomarkers due to their controlling role in disease development [13]. Noncoding RNAs play vital regulatory roles in multiple biological processes.

miRNAs are small noncoding ribonucleic acid molecules with a length of 20-22 base pairs that play a vital role in regulating gene expression at the posttranscriptional level by either blocking mRNA translation or inducing mRNA degradation. In some cases, miRNAs may enhance the transcription of a gene or translation of an mRNA, thereby increasing the level of the protein product [14]. Accumulating evidence reveals the importance of miRNAs in regulating key signaling and lipid homeostasis pathways that alter the balance between the progression and regression of atherosclerotic plaques [15]. Importantly, miRNAs are not only associated with lipoprotein metabolism but are also implicated in the regulation of endothelial cell inflammation and plaque progression [16, 17]. In addition, miRNAs regulate leukocyte recruitment and activation in atherosclerosis, one of the earliest pathogenic events in atherosclerosis [17].

According to the classification of ncRNA, lncRNAs are ncRNAs that are more than 200 base pairs in length. Although the function of most lncRNAs remains unknown, it has become clear that these molecules are intimately involved in various biological processes. lncRNAs can regulate gene expression programs through a variety of mechanisms, such as epigenetic modifications of DNA, alternative splicing, posttranscriptional gene regulation and mRNA stability and translation [18, 19]. Given their established roles in transcriptional regulation, lncRNAs play a key role in numerous cellular events including proliferation, migration, apoptosis and development [18]. lncRNAs are now known to regulate the expression of protein-coding genes: they can positively or negatively control the expression of their target genes. Several lncRNAs are involved in *cis* inactivation of larger genomic regions by epigenetic mechanisms. More recently, it was found that lncRNAs can act in *cis* to regulate the expression of neighboring genes during cardiomyocyte differentiation. Notably, many lncRNAs are now known to regulate the expression of genes by a*trans* mechanism. These molecules can act as scaffolds, bringing together multiple proteins to form ribonucleoprotein complexes. In addition to their role in chromatin regulation, lncRNAs can also function as molecular “decoys” for transcription factors and other regulatory proteins. Finally, lncRNAs may exert their biological activity through their ability to act as endogenous decoys for miRNAs.

Many researchers have reported that miRNAs and lncRNAs play an important role in the pathology of CAD [20, 21]. Specific miRNAs or lncRNAs were evaluated not only as diagnostic markers but also as predictors of future cardiovascular events in CAD patients.

## The pathophysiologic role of miRNAs in CAD development

Atherosclerosis and aberrant thrombosis are the primary pathological changes involved in CAD. The onset of atherosclerosis is partly mediated by the dysfunction of ECs, and the migration and proliferation of vascular smooth muscle cells (VSMCs) [3]. Meanwhile, numerous immune cells and relevant cytokines in plaque constantly recruit leukocytes, promote cell apoptosis, influence plaque composition and finally destabilize and exacerbate lesion development [22, 23]. This multifactorial process is ascribed to the interactions of several key components, including lipoproteins, inflammatory cells and vascular cells.

An increasing number of studies have identified that miRNAs regulate EC, SMC, and macrophage function; vascular inflammation; and metabolism, suggesting the possibility that miRNAs influence the progression of CAD (Table 1). The levels of circulating miRNAs reflect pathological conditions, and some miRNAs have been identified as novel biomarkers with the potential to detect atherosclerosis or CAD at its earliest stages in clinical practice [24].

**Table 1:** Summary of coronary artery disease-related miRNAs.

Classifications	MiRNAs	Cell type/process	Dysfunction type	Expression	Functions	Refs
ECs/VSMCs function	miR-126-5p	Plasma/ ECs	Regulation	Down-regulated	play the role of anti-atherogenisis and enhance ECs repair	[27, 28]
	miR-17-92 cluster (miR-17, miR-18a, miR-20a, miR-19a/b)	Plasma/ ECs	Regulation	Down-regulated	attenuate TNF-α-induced endothelial cell apoptosis	[29, 30]
	miR-206	VSMCs/ plasma	Regulation	Up-regulated	anti-atherosclerosis by inhibiting the expression of FOXP1 in VSMCs	[31, 32]
	miR-574-5p	VSMCs/ plasma	Expression	Up-regulated	promote cell proliferation and inhibits apoptosis by inhibiting ZDHHC14 gene expression	[34]
	miR-23a	ECs	Regulation	Down-regulated	suppress cellular migration and vasculogenesis via targeting EGFR	[35, 37]
	miR-21	VSMCs	Regulation	Up-regulated	promote aberrant VSMCs proliferation	[35, 36]
	miR-361-5p	Plasma/ ECs	Regulation	Up-regulated	suppress VEGF expression and EPCs activity	[37]
Inflammatory	miR-146a	ECs	Regulation	Up-regulated	inhibit the expression of adhesion molecules and inflammatory cytokines	[40, 41]
	miR-10a	ECs	Regulation	Down-regulated	regulate inflammatory responses	[42, 43]
	miR-155	Macrophages/ SMCs	Regulation	Down-regulated	function as anti-angiogenesis via suppression of AT1R and promote inflammatory signal transduction	[44, 45]
	miR-22	PBMCs	Regulation	Down-regulated	anti-inflammatory response by targeting MCP-1	[46]
Lipid metabolism	miR-486, miR-92a	Plasma/ ECs	Regulation	Up-regulated	participate in HDL biogenesis	[47, 48]
	miR-24, miR-103a	PBMCs	Expression	Up-regulated	participate in cholesterol synthesis/transport and fatty acid metabolism	[49]
	miR-208a	Plasma/ ECs	Regulation	Up-regulated	regulate cardiac hemostasis and lipid metabolism	[51]
	miR-370, miR-122	Plasma	Regulation	Up-regulated	regulate cholesterol and fatty-acid metabolism	[51, 52]
	miR-93	Serum/ ECs	Regulation	Up-regulated	regulate serum cholesterol level via targeting ABCA1	[52]
	miR-33a	Serum/ ECs macropahge	Regulation	Up-regulated	regulate cholesterol accumulation by affecting HDL biogenesis	[50]
	miR-17-5p	Plasma/ macropahge	Expression	Up-regulated	attenuate atherosclerotic lesion by mediating autophagy pathway and regulate cholesterol efflux	[53, 54]
Platelet function	miRNA-223, miRNA-197	Serum	Regulation	Up-regulated	regulate thrombocyte activation	[55, 56]
	miR-624*, miR-340*	Platelet	Expression	Up-regulated	govern platelet reactivity	[57]
Circulating miRNAs	miR-126	Circulating MVs	Regulation	Down-regulated	regulate the proliferation and migration of ECs	[25]
	miR-199a	Cardiomyocyte/ MVs	Regulation	Down-regulated	act as a suppressor of cardiomyocyte autophagy	[25]
	miR-222	Endothelial MPs	Regulation	Down-regulated	anti-inflammatory by inhibiting ICAM-1 expression	[63]
	miR-149, miR-424	Plasma	Expression	Down-regulated	inhibit pro-inflammatory-induced angiogenesis	[58, 59]
	miR-765	Plasma/ ECs	Expression	Up-regulated	influence arterial stiffness through modulating apelin expression	[58]
	miR-487a	Serum	Expression	Up-regulated	involve in the occurrence of atherosclerosis by regulating TAB3 expression	[61]
	miR-502	Serum	Expression	Up-regulated	suppress autophagy process and play an atheroprotective role	[61]
	miR-215	Serum/ ECs	Expression	Up-regulated	stimulate neointimal lesion formation	[61]
	miR-29b	Serum/ ECs	Expression	Down-regulated	regulate myocardial ischemia and cardiac remodeling	[62]
	miR-145	Plasma/VSMCs	Regulation	Down-regulated	play a role in VSMCs phenotypic modulation	[44]
	let-7c	Plasma/ECs	Regulation	Down-regulated	regulate cell differentiation and promote ECs apoptosis by inhibiting of Bcl-xl	[44. 60]

### MiRNAs regulate the function of vascular ECs and VSMCs

ECs and VSMCs constitute the main cell types within the vasculature. The vascular endothelium is the natural barrier between the circulating blood and the subendothelial tissues of the vascular wall. Dysfunctional ECs play a pivotal role in atherosclerosis by impairing endothelium-dependent vasodilatation, increasing vascular permeability, upregulating the expression of chemokine and adhesion molecules and triggering platelet activation and aggregation [25]. VSMCs migrate from the arterial media into the intima, where they proliferate and produce the extracellular proteins that constitute atherosclerotic plaques. VSMCs can modulate their phenotype in response to environmental stimuli via a process characterized by decreased gene expression of VSMC contractile markers and increased proliferation, migration, and extracellular matrix synthesis. Such a phenotypic switch represents one of the principal cellular events underlying various VSMC-related pathological conditions, including hypertension, atherosclerosis, postangioplasty restenosis, and angiogenesis.

Accumulating studies reveal the importance of miRNAs in regulating the proliferation and migration of local ECs, the mobilization of endothelial progenitor cell (EPC) and the promotion of VSMC migration and activation. For example, miR-126-5p plays a crucial antiatherogenic role by regulating the function of ECs and enhancing endothelial repair. The expression of plasma miR-126-5p was significantly lower in CAD patients [26]. The decreased level of miR-126-5p expression is critically involved in the regulation of plaque formation. In addition, miR-126-5p negatively regulates leukocyte adherence to ECs by targeting endothelial vascular cell adhesion molecule-1 (VCAM-1) [27].

The miR-17-92 cluster, which has been reported to be significantly downregulated in patients with CAD, is closely associated with TNF-α-induced apoptosis in endothelial cells [28]. For example, miR-19b plays a critical role in the attenuation of TNF-α-induced endothelial cell apoptosis, and this function is closely linked to the Apaf1/caspase-dependent pathway. The downregulation of miR-19b alleviated its inhibition of TNF mRNA expression and resulted in increased TNF-α expression, which subsequently resulted in reduced miR-19b expression [29]. This finding may provide new insight into the clinical application of endothelial repair in the setting of CAD.

miR-206 and miR-574-5p were markedly upregulated in CAD patients compared with control subjects [30]. miR-206 was considered to regulate the activities of EPCs by targeting the protein kinase PIK3C2α, which showed decreased expression in CAD EPCs [31]. Furthermore, miR-206 may be a protective factor in atherosclerosis by inhibiting the expression of FOXP1, which is involved in controlling the proliferation of VSMCs [32]. In humans, miR-574-5p was regarded to be hosted by the first intron of the gene encoding Noxp20 on chromosome 4, and the expression of miR-574-5p has been found to be associated with CAD. miR-574-5p promotes proliferation and inhibits apoptosis in VSMCs by inhibiting ZDHHC14 gene expression, suggesting that miR-574-5p is a CAD-related factor that could be used as a molecular target in CAD treatment [33].

Some miRNAs could promote VSMC migration and activation, which alters the progression of plaques in CAD. For example, miR-21 and miR-23a might serve as biomarkers of CAD development and progression. Studies have shown that miR-21 was elevated and miR-23a was decreased in CAD patients [34]. miR-21 was demonstrated to have a role in the aberrant proliferation of VSMCs, and inhibition of miR-21 reduced the proliferation and increased the apoptosis of VSMCs [35]. In addition, it has been reported that the level of miR-21 was also elevated in CAD patients, which indicates that miRNAs are important as potential biomarkers of atherosclerosis progression and, consequently, CAD progression. miR-23a suppresses EPC activities in CAD patients via targeting epidermal growth factor receptor (EGFR). The overexpression of miR-23a suppressed cellular migration and vasculogenesis to normal levels. In addition, the level of miR-361-5p was also dramatically increased in CAD patients [36]. It has been reported that the abundance of miR-361-5p secreted by CAD-EPCs was increased in CAD patient plasma. This miRNA was poised to suppress vascular endothelial growth factor (VEGF) expression and EPC activities via targeting VEGF, indicating that the level of miR-361-5p in plasma from diseased individuals should be evaluated as a potential novel diagnostic biomarker for CAD.

### Inflammation-related miRNAs in CAD

It is widely considered that vascular inflammation and leukocytes activation are associated with all stages of atherosclerosis [37]. Vascular endothelial inflammation and associated endothelial dysfunction represent critical initial steps in atherosclerosis and CAD [25, 38]. When stimulated by atherosclerotic factors, ECs express adhesion and chemoattractant molecules that recruit inflammatory monocytes into the vascular wall. Recently, more miRNAs have been reported to be involved in the regulation of the immune system, demonstrating that miRNAs modulate the immune responses involved in CAD.

miR-146a, which was originally identified as an inflammation-related miRNA, acts to restrain and decrease the duration of endothelial activation in response to proinflammatory cytokine stimulation. The overexpression of miR-146a, in turn, inhibits adhesion molecules such as ICAM-1, VCAM-1, and E-selectin, and it also inhibits the expression of other genes related to inflammation. The level of miR-146a is decreased in CAD patients with poor coronary collateral circulation, indicating that it is a predictor of CAD [39, 40].

miR-10a expression has been found to be lower in the atherosusceptible regions of the inner aortic arch and aortorenal branches than in other regions and to regulate inflammatory responses in atherosclerosis susceptible endothelium [41, 42]. Plasma miR-10a levels were significantly downregulated in CAD patients compared with non-CAD controls.

miR-155 has recently emerged as a novel component of inflammatory signal transduction in atherosclerosis and is considered a mechanosensitive athero-miRNA [43]. The expression of miR-155 is upregulated in atherosclerotic lesions of humans and mice, predominantly in pro-inflammatory macrophages and SMCs where it targets BCL6 (B-cell lymphoma 6) and HMGB1 (high mobility group box 1) [44]. The expression of miR-155 was significantly reduced in patients with CAD compared to controls [43].

Studies have shown that some peripheral blood mononuclear cell (PBMC)-derived miRNAs may participate in the inflammatory response. For instance, miR-22 is downregulated in PBMCs from patients with CAD; and in addition, miR-22 may participate in inflammatory responses by targeting MCP-1, therefore contributing to CAD [45]. The overexpression of miR-22 significantly repressed MCP-1 expression at both the mRNA and protein levels in PBMCs, whereas the inhibition of miR-22 showed the opposite effects.

### miRNAs involved in lipid metabolism in CAD

miR-486 and miR-92a, in association with some high-density lipoprotein (HDL) components, can identify vulnerable CAD patients [46]. miR-486 and miR-92a presented the highest expression in CAD sera compared with normal sera. Studies have shown that increased levels of miR-486 in HDL2 and miR-92a in HDL3 were particularly associated with vulnerable CAD patients’ sera. Furthermore, miR-92a has a pro-atherosclerotic function and is involved in endothelial dysfunction [47].

Additionally, the expression levels of miR-24, miR-33a, miR-103a, and miR-122, which have been reported to be associated with lipid metabolism in both patients and animal models, were significantly increased in the PBMCs of CAD patients [48]. Several key genes involved in cholesterol synthesis/transport and fatty acid metabolism were considered to be regulated by miR-33 [49]. MiR-103a could increase lipolysis and elevate circulating free fatty acid levels by the repression of caveolin-1.

miR-208a, miR-370 and miR-122 are fundamental molecules in cardiac hemostasis or lipid metabolism [50]. Study show that anti-miR-208a treatment prevented pathological cardiac remodeling and improved cardiac function and survival during hypertension-induced heart failure in Dahl hypertensive rats. miR-370, as a key miRNA in lipid metabolism could downregulate the expression of the carnitine palmitoyltransferase 1α gene that controls fatty acid oxidation [51]. miR-122 is a central regulator of cholesterol and fattyacid metabolism, and miR-122 silencing leads to a decrease in plasma cholesterol levels in experimental animals. The expression levels of these three miRNAs were much higher in CAD patients than in subjects without CAD [51].

Furthermore, a significant reduction in miR-33a and miR-93 levels was found in coronary patients. Upregulated serum miR-93 is positively correlated with increased serum cholesterol level in coronary atherosclerosis patients via the targeting ABCA1 [51]. In addition, miR-33a regulates cholesterol accumulation by affecting HDL biogenesis.

MiR-17-5p, an atherogenic agent, was detected at a higher level in CAD plasma [52]. The knockdown of miR-17-5p attenuates the atherosclerotic lesion area in apolipoprotein E-deficient mice. It has been reported that interferon-stimulated gene 15 (ISG15) promotes cholesterol efflux by activating autophagy via the miR-17-5p/Beclin-1 pathway in THP-1 macrophage-derived foam cells [53]. Thus, miR-17-5p may function as a useful biomarker with high sensitivity and specificity in the diagnosis of coronary atherosclerosis in the initial and developmental stages.

### Association of miRNAs with platelet function

The levels of miR-197 and miR-223 were evaluated in serum of CAD patients and were strongly correlated with platelet activation [54]. Platelet-derived miR-197 levels are associated with thrombocyte activation and were found to be altered in patients receiving antiplatelet therapy indicating a potential role for this miRNA in CAD progression. miR-223 is deemed to be associated with platelet activation. Activated platelets release microparticles containing functional complexes of miR-223 with the Argonaute 2 protein (Ago2) that can be internalized by recipient cells [55]. Furthermore, miR-223 has been exposed as a moderator of endovascular high-density lipoprotein-associated anti-inflammatory effects, which are involved in the regulation of multiple inflammatory genes as well as in cholesterol homeostasis.

Two platelet-derived miRNAs, miR624* and miR340*, were significantly upregulated in patients with CAD compared to healthy controls [56]. These platelet-derived miRNAs can potentially fine-tune the expression of specific gene products that may be involved in governing platelet reactivity. Therefore, a dysfunctional miRNA-based regulatory system could lead to the development of acute platelet-related cardiovascular diseases.

### Circulating miRNAs in microparticles or plasma as biomarkers in CAD

Circulating miRNA levels may reflect pathological conditions, and some circulating miRNAs have been recognized as novel biomarkers with the potential for the early diagnosis of CAD.

Circulating miR-149 and miR-424 were downregulated, whereas miR-765 was upregulated, in middle-aged patients, indicating that these miRNAs might be noninvasive biomarkers for the diagnosis of CAD [57]. miR-424 has been found to inhibit inflammatory-induced angiogenesis through the direct targeting of CD40 [58]. miRNA-765 influences arterial stiffness through modulating apelin expression.

Circulating miR-145 and let-7c might also be diagnostic biomarkers for coronary artery disease [43]. The plasma level of vascular SMC-enriched miR-145 was significantly downregulated in patients with CAD compared to control subjects. miR-145 plays a critical role in VSMC phenotypic modulation in abnormal vascular pathologies. miR-145 is downregulated in human plaque atherosclerosis and aneurysms, and in animal models of vascular injury. The let-7 family has been discovered to play important roles in both cardiovascular biology and disease. The overexpression of let-7c enhances endothelial cell apoptosis through the inhibition of Bcl-xl a direct target of let-7c, suggesting a proatherosclerotic role for let-7c. In addition, let-7c could regulate cellular functions associated with macrophage phenotypes by promoting M2 polarization and suppressing M1 activation through the regulation of the transcription factor C/EBP-δ [59].

The levels of circulating miR-487a, miR-502, miR-208 and miR-215 in serum were significantly increased, whereas the level of miR-29b was markedly decreased, in atypical coronary artery disease patients compared with normal controls [60]. miR-215 has been reported to stimulate subclinical atherosclerosis via neointimal lesion formation through promoting β-catenin activation and upregulating α-smooth muscle actin (a-SMA). miR-487a may be involved in the occurrence of atherosclerosis by regulating TAB3 expression. miR-29b and miR-208 were predicted to target numerous extracellular matrix genes and to play a role in myocardial ischemia and cardiac remodeling [61]. miR-502 could suppress the autophagy process and play an atheroprotective role by directly targeting RAB1B and AP2B1. These findings suggest that circulating miRNAs may serve as potential diagnostic biomarkers in CAD patients.

Recently, microvesicles (MVs) containing cargo such as mRNAs, miRNAs, receptors, and specific proteins from the parent cell, have been reported to facilitate the transfer of circulating miRNA between cells. MV-associated miRNAs may contribute to intercellular signaling mechanisms. Studies have shown that MV-associated but not their freely circulating counterparts predict the occurrence of cardiovascular events in patients with stable coronary artery disease. Platelet-derived miR-126 was identified to influence angiogenesis via the regulation of the proliferation and migration of endothelial cells. The expression of MV-associated-miR-126 and MV-associated-miR-199a was significantly reduced in patients with CAD compared with healthy subjects [24].

Furthermore, endothelial microparticles (EMP) contain and transfer miR-222 into endothelial target cells and inhibit ICAM-1 expression, which contributes to vascular inflammation and atherosclerosis. A study showed that miR-222 levels were significantly reduced in CAD patients compared to patients without CAD [62]. miR-19b-containing EMP potentially have proatherosclerotic effects that involve the aggravation of inflammation in perivascular adipose tissue (PVAT) by the targeting of SOCS3. These EMP may be regarded as a new therapeutic target for the prevention and treatment of atherosclerosis [63]. These findings suggest that these circulating miRNAs may be used as novel diagnostic markers for CAD.

## Roles of lncRNAs in CAD

Recent studies suggest critical roles for lncRNAs in modulating the initiation and progression of cardiovascular diseases.

Many lncRNAs have been shown to be functional and to be involved in specific physiological and pathological processes through epigenetic and transcriptional or posttranscriptional regulatory mechanisms [64]. Several studies have shown that lncRNAs are involved in the development of various CVDs, including heart failure, cardiac hypertrophy, cardiometabolic diseases, myocardial infarction, and atherosclerosis [21, 65]. Recent studies have indicated that lncRNAs are involved in the atherosclerosis-related regulation of smooth muscle cells, endothelial cells, macrophages and lipid metabolism, indicating the potential function of lncRNAs in CAD development (Table 2). Thus, novel lncRNAs may have a use as biomarkers for CAD.

**Table 2:** Summary of coronary artery disease-related lncRNAs.

Classifications	LncRNAs	Celltype/process	Action mode	Expression	Functions	Refs
**ECs/VSMCs function**	ANRIL	VSMC	Antisense	Down-regulated	regulate expression of CDKN2B and modulate VSMCs proliferation	[67, 68]
	H19	PBMCs/ VSMCs/Plasma	Antisense	Up-regulated	function as a sponge of the let-7 to protect VSMCs	[69-71]
	HIF1a-AS1	ECs/VSMCs	Antisense	Up-regulated	partake in process of atherosclerosis through controlling VSMCs and ECs apoptosis	[72, 73]
	LincRNA-p21	ECs/VSMCs	LincRNA	Down-regulated	a novel regulator of neointima formation, vascular smooth muscle cell proliferation, apoptosis, and atherosclerosis by enhancing p53 activity	[74, 75]
	RNCR3	ECs/ VSMCs	LincRNA	Up-regulated	negatively regulate hypercholesterolemia and inflammatory factor releases, suppress apoptosis of ECs and VSMCs	[76]
	TGFB2-OT1	ECs/VSMCs	ceRNA	Up-regulated	regulate autophagy in ECs and VSMCs	[77]
	Lnc-Ang362	VSMCs	Intronic lncRNA	Up-regulated	coregulate in response to Ang II to regulate VSMCs proliferation	[78]
	HAS2-AS1	VSMCs	Antisense	Up-regulated	stabilize or augment the expression of HAS2 mRNA involved in neointimal hyperplasia and inflammation	[79]
	SMILR	VSMCs	lincRNA	Up-regulated	remodel of the extracellular matrix and neointimal formation, and inflammation	[80]
	SENCR	ECs	Antisense	Down-regulated	regulate endothelial differentiation and angiogenesis	[81]
	MEG3	ECs	Intronic lncRNA	Down-regulated	regulate endothelial differentiation	[82]
**Inflammatory**	LincRNA-Cox2	ECs	lincRNA	Up-regulated	promote inflammatory gene transcription in macrophages	[84]
	MKI67IP-3	ECs	ceRNA	Down-regulated	negatively regulate let-7e to regulate endothelial function and inflammation	[85]
	LncRNA-lethe	Macrophages	ceRNA	Down-regulated	negatively regulate NF-kB expression	[86]
**Lipid metabolism**	LincRNA-DYNLRB2-2	Macrophages	lincRNA	Up-regulated	promote ABCA1-mediated inflammation and cholesterol efflux in foam cells	[87]
	RP5-833A20.1	Macrophages	Antisense	Down-regulated	regulate cholesterol homeostasis and inflammatory reactions through inhibit NFIA expression	[88]
	APOA1-AS	Plasma	Antisense	Up-regulated	inhibit the expression of APOA1 and the formation of HDL	[89]
	HOTAIR	Macrophages/ ECs	Antisense	Down-regulated	attenuate the suppression of cell viability and enhancement of cell apoptosis caused by ox-LDL	[91, 92]
**Circulating lncRNAs**	CoroMarker	Plasma/ Macrophages	LncRNA	Up-regulated	decrease pro-inflammatory cytokine secretion from THP-1 monocytic cells	[93, 94]
	LncPPARδ	PBMCs	LncRNA	Up-regulated	regulate the expression of PPARδ to mediate inflammatory response	[95]
	LIPCAR	Plasma	Mitochondrial lncRNA	Up-regulated	regulate mitochondrial pathways of oxidative phosphorylation and inflammasome activation	[71, 96]

### lncRNAs modulate the functions of ECs and VSMCs

It has been indicated that many lncRNAs may regulate endothelial functions, especially angiogenesis. lncRNAs are key molecular players in vascular EC proliferation and VSMC migration; thus, the dysregulated expression of specific lncRNAs has been reported to contribute to CAD.

Studies have shown that ANRIL regulates VSMC growth through CDKN2A/B (known as p16^INK4A^/p15^INKB^), which participates directly in the pathogenesis of atherosclerosis [66]. In atherosclerotic lesions, there are decreased levels of *p*15^INK4b^ expression and increased levels of VSMC proliferation, suggesting that the 9p21 locus is susceptible to SNPs that influence the transcription of the long noncoding RNA ANRIL, which overlaps the region. These SNPs increase the expression of ANRIL transcripts in both plaque and peripheral blood. Knockdown of ANRIL in VSMCs increases the expression of CDKN2B and inhibits VSMC proliferation. Thus, ANRIL is closely correlated with the severity of atherosclerosis. Future studies may concentrate on circulating ANRIL, which is a potential atherosclerosis risk factor and therapeutic target, as a biomarker of CAD. Furthermore, ANRIL was shown to be highly expressed in atherosclerotic plaques and might be a “fine-tuner” within the inflammatory NF-κB pathway by acting as an antisense regulator of the CDKN2B-CDKN2A gene cluster at the 9p21 locus [67].

The lncRNA-H19 is highly expressed in the neointima after injury and in human atherosclerotic lesions but is barely expressed in normal arteries. H19 lncRNA functions as a sponge of let-7 family miRNAs, and let-7 miRNAs have been indicated to protect VSMCs from oxidative damage [68, 69]. The level of H19 in plasma is elevated in a Chinese population with CAD. Furthermore, individuals carrying the risk alleles of H19 display a higher risk of coronary artery disease. The data showed that the plasma level of H19 was increased in CAD patients with heart failure compared to those with normal cardiac function. Thus, H19 is associated with a risk of CAD and may be considered a novel biomarker for CAD [70].

The lncRNA HIF-1 alpha-antisense RNA 1 (HIF1a-AS1) participates in the process of atherosclerosis through controlling VSMC and EC apoptosis [71]. Suppression of the lncRNA HIF1A-AS1 in VSMCs reduced cell apoptosis and promoted cell proliferation, indicating that HIF1A-AS1 plays a major role in the pathophysiology of VSMCs. In addition, the expression of HIF1A-AS1 was considered to be regulated by BRG1 in VSMCs [71]. Previous studies suggested that BRG1 plays an important role in regulating cardiac growth, differentiation and gene expression. BRG1 overexpression can promote apoptosis and reduce proliferation in VSMCs. The lncRNA HIF1A-AS1 may play a significant role in the pathogenesis of CVD induced by hyperlipidemia, such as CAD [72].

Clinical findings also reveal that coronary tissues from CAD patients exhibit a decreased level of lincRNA-p21 compared to aortic tissues from non-CAD patients [73]. lincRNA-p21 has been recently established to repress the proliferation and induce the apoptosis of VSMCs and mouse macrophage cells in vitro. Studies have identified that lincRNA-p21 is a novel regulator of neointima formation, vascular smooth muscle cell proliferation, apoptosis, and atherosclerosis by its effects on enhancing p53 activity [74]. These results suggest that lincRNA-p21 may also serve as a potential biomarker of CAD and as a therapeutic target. Furthermore, polymorphisms in lincRNA-p21 have been associated with CAD risk. The therapeutic potential of lincRNA-p21 in acute vascular injury is suggested by its regulatory role in cell proliferation and apoptosis in CAD.

lncRNA-RNCR3, also known as LINC00599, is a long intergenic nonprotein coding RNA, and its expression is significantly upregulated in ECs and VSMCs from mouse and human aortic atherosclerotic lesions [75]. RNCR3 knockdown accelerates the development of atherosclerosis, aggravates hypercholesterolemia and the release of inflammatory factors, and reduces the proliferation and migration but accelerates the apoptosis of ECs and VSMCs. Compared with control mice, mice in which RNCR3 was downregulated with shRNA demonstrated aggravated atherosclerosis in thoracic aortic tissue and increased inflammatory factors in plasma. In vitro treatment with oxidized low-density lipoprotein (ox-LDL) increased RNCR3 levels in human umbilical vein endothelial cells (HUVECs) and VSMCs, reducing proliferation and viability and increasing apoptosis. A recent in vitro study demonstrated that exosomes derived from ECs are rich in RNCR3, which is transferred to VSMCs and induces their proliferation and migration. The proposed mechanism of action in ECs is that RNCR3 regulates the transcription factor KLF2 by sponging miR-185-5p, which targets KLF2. Thus, because of the atheroprotective role of RNCR3 in atherosclerosis, its induced upregulation potentially represents a therapeutic intervention.

TGFB2-OT1 (TGFB2 overlapping transcript 1) is a newly discovered lncRNA derived from the 3’ UTR of TGFB2, which can regulate autophagy in VSMCs. The level of TGFB2-OT1 was markedly increased by exposure to lipopolysaccharide and oxidized low-density lipoprotein [76]. TGFB2-OT1 promotes autophagy via ATG3 and ATG7 and participates in inflammation via elevating the level of SQSTM1. TGFB2-OT1 is associated with autophagy and inflammation in vascular endothelial cells as a ceRNA that bind to miR-3960, miR-4488 and miR-4459, which targets CERS1, NAT8L, and LARP1, respectively.

The dysregulation of angiotensin II (Ang II) activity can lead to atherosclerosis and hypertension. Many of the lncRNAs are located proximal to other Ang II-regulated genes, suggesting that they may be coregulated in response to Ang II. lnc-Ang362 is the host transcript for miR-221 and miR-222, which have been previously associated with the regulation of VSMC proliferation and neointimal hyperplasiain vascular diseases including coronary heart disease and atherosclerosis [77].

Extracellular matrix remodeling and neointimal formation are additional fundamental features of CAD. The deposition of hyaluronan, which is synthesized by HAS2, contributes to this process. Two lncRNAs independently regulate HAS2: HAS2-AS1 (HAS2 antisense RNA 1) and SMILR. The antisense transcript HAS2-AS1 was increased in atherectomy samples collected from severely diseased carotid arteries and appears to promote HAS2 transcription in VSMCs in the presence of O-GlcNAcylation by altering chromatin configuration [78]. SMILR was significantly upregulated in VSMCs stimulated with interleukin 1α and platelet-derived growth factor compared to unstimulated cells. In addition, the level of SMILR in plasma correlated with the level of the inflammatory marker C-reactive protein and was elevated in unstable atherosclerotic plaques from patients [79].

SENCR, a vascular-enriched long noncoding RNA, is a member of ETS family, and has been described as an early regulator of hemato-endothelial development. SENCR contributes to the regulation of endothelial differentiation from pluripotent cells and controls the angiogenic capacity of HUVECs. SENCR expression is down regulated in patients with EC dysfunction and atherosclerotic vascular disease [80].

It has been demonstrated that the expression of lncRNA maternally expressed gene 3 (MEG3) was downregulated in CAD tissues compared to control tissues [81]. The elevated expression of MEG3 suppressed miR-21 expression in the ECs and promoted the expression of RhoB and PTEN, which are the direct target genes of miR-21. Moreover, miR-21 expression was inversely correlated with MEG3 expression in CAD tissues. Thus, SENCR and MEG3 may be novel biomarkers for the regulation of endothelial development and function involved in CAD pathogenesis.

### Inflammation-related lncRNAs in CAD

Atherosclerosis and CAD are partially caused by chronic lipid-induced inflammatory disease of the vessel wall, which is characterized by innate and adaptive immune system involvement. Vascular inflammation and leukocyte activation are widely considered to be associated with all stages of atherosclerosis and CAD. lncRNAs play a role in regulating the release of pro-inflammatory cytokines in monocytes and are probably implicated in innate immunity during the pathology [82].

lincRNA-Cox2 is one of the most highly induced lncRNAs in studies examining the innate immune response. lincRNA-Cox2 could promote late inflammatory gene transcription in macrophages through modulating SWI/SNF-mediated chromatin remodeling [83].

Lnc-MKI67IP-3, which acts as a sponge or a competing endogenous RNA (ceRNA) for let-7e, plays an important role in the inflammatory responses of VECs and the development of atherosclerosis [84]. Moreover, the levels of let-7e, lnc-MKI67IP-3 and IκBβ were also abnormal in oxLDL-treated VECs and atherosclerotic plaques. These findings demonstrated that this molecule might play an important role in the inflammatory responses of VECs and the progression of atherosclerosis.

Studies have shown that lncRNAs may be critical mediators of NF-κB-regulated gene transcription and may participate in the pathogenesis of various inflammatory diseases. Lethe, a pseudogene lncRNA, is selectively induced by proinflammatory cytokines via stimulation with NF-κB or glucocorticoid receptor agonists and functions in negative feedback signaling to NF-κB [85]. The Lethe has been defined as exhibiting an anti-inflammatory effect by binding to the p65 subunit of NF-κB. Lethe level decreases with organismal age, creating a physiological state associated with increased NF-κB activity. Furthermore, studies have shown that Lethe is involved in the regulation of ROS production in macrophages through the modulation of NOX2 gene expression via NF-κB signaling. These findings suggest that the expression of Lethe plays a critical role in the regulation of atherosclerosis- and CAD- related processes that involve inflammation.

### lncRNAs with a function in lipid metabolism

It is well known that lipid imbalance is a significant risk factor of atherosclerosis. Numerous pro-atherogenic and antiatherogenic properties are attributed to oxidized LDL (Ox-LDL). The incubation of macrophages with Ox-LDL but not with native LDL leads to cholesterol ester accumulation. Recent studies have indicated that lncRNAs are involved in lipid metabolism.

Studies have demonstrated that Ox-LDL significantly induced lincRNA-DYNLRB2-2 expression, which promoted ABCA1-mediated cholesterol efflux and inhibited inflammation through G protein-coupled receptor 119 (GPR119) in acute monocytic leukemia (THP-1) macrophage derived foam cells [86]. Another long noncoding RNA, RP5-833A20.1, is located in intron 2 of the NFIA gene and is an antisense lncRNA of NFIA [87]. This study demonstrated that lncRNA RP5-833A20.1 could attenuate ABCA1 levels, thus reducing cholesterol levels. This lncRNA increases inflammatory cytokines and reduces cholesterol efflux via the miR-382-mediated NFIA pathway. The RP5-833A20.1/hsa-miR-382-5p/NFIA pathway was critical in the regulation of cholesterol homeostasis, inflammatory responses, and foam cell formation.

Apolipoprotein A-1 (APOA1) is the major protein component of plasma high-density lipoprotein (HDL), which plays an important role in protecting against cardiovascular disease. APOA1-AS is a long noncoding antisense transcript that acts as a negative transcriptional regulator of APOA1. APOA1-AS has been demonstrated to negatively regulate APOA1 expression both in vitro and in vivo, making APOA1-AS a potential therapeutic target for the treatment of cardiovascular disease [88].

Accumulating evidence has indicated that some members of the family of HOX genes exert significant regulatory effects on the cardiovascular system. The expression level of lncRNA HOXC cluster antisense RNA 1 (HOXC-AS1) and HOXC6 were downregulated in atherosclerotic plaques. In addition, ox-LDL decreased the expression of HOXC-AS1 and HOXC6 in a time-dependent manner, and HOXC-AS1 could suppress ox-LDL-induced cholesterol accumulation through promoting HOXC6 expression in human THP-1 macrophages [89]. These findings indicate that HOXC-AS1 may participate in regulating the inflammatory response in CAD, but this hypothesis needs to be further verified. HOTAIR, another important lncRNA transcribed from the HOXC locus, was initially described to downregulate HOXD gene expression through the recruitment of polycomb repression complex 2 (PRC2) in order to trimethylate H3K2. HOTAIR expression was found to be much lower in ECs from atherosclerotic plaques than in normal ECs. HOTAIR plays a protective role in EC injury. HOTAIR could be suppressed by ox-LDL treatment in a dose- and time-dependent manner. The overexpression of HOTAIR significantly attenuated the suppression of cell viability and enhancement of cell apoptosis caused by ox-LDL [90]. Furthermore, HOTAIR upregulation in the cardiomyocytes of mice with LPS-induced sepsis promoted TNF-α production in the circulation by activating NF-κB by involving the phosphorylation of the NF-κB p65 subunit [91]. Moreover, HOTAIR silencing in mice could protect the myocardium from LPS-induced myocardial dysfunction. Further studies are needed to explore the potential mechanism of HOXC-AS1 and HOTAIR in CAD.

### Circulating lncRNAs as biomarkers in CAD

Many studies have indicated that circulating PBMCs which constitute 5-10% of peripheral blood leukocytes, play a pivotal role in the development of CAD by migrating into the arterial wall and increasing the size of atherosclerotic plaques. CoroMarker, a circulating monocyte-derived lncRNA, is a novel biomarker with high sensitivity and specificity for the diagnosis of CAD. CoroMarker plays an important role in inflammatory processes involved in atherogenesis. The downregulation of CoroMarker has been shown to decrease proinflammatory cytokine secretion from THP-1 monocytic cells. The level of CoroMarker was significantly increased in CAD patients compared with non-CAD subjects [92, 93].

lncPPARδ can be a useful biomarker for CAD, especially when combined with risk factors. lncPPARδ regulates the expression of a neighboring protein-coding gene, PPARδ, and its direct target genes, ADRP and ANGPTL4 [94]. Furthermore, lncPPARδ expression was sharply increased in PBMCs in CAD, indicating that lncPPARδ plays a role in regulating the inflammatory response of PBMCs.

LIPCAR was characterized as a mitochondrial lncRNA with a potential utility in predicting death in patients with heart failure. The level of LIPCAR was reported to be upregulated in patients with chronic HF independent of pathogenesis, and a higher LIPCAR level was associated with a higher risk of cardiovascular death. It was demonstrated that higher LIPCAR levels are strongly associated with cardiovascular mortality in patients with HF and that LIPCAR measurement provides independent information that augments classic risk stratification [95]. A recent study indicated that the plasma level of LIPCAR is increased in CAD patients with heart failure compared to those with normal cardiac function. An increased plasma levels of LIPCAR may be regarded as a novel biomarker for CAD [70].

## miRNA-lncRNA interaction in disease

The suggestion of numerous interactions between the different classes of RNA due to complementary binding sites is becoming increasingly clear. miRNAs might thus directly control the action and transcription of lncRNAs, whereas the effects of this control might in turn result in the smothering of miRNA-related effects by endo-sponge activity. These closely interwoven relationships emphasize the likely involvement of a network, rather than a single gene; this distinction is important when investigating changes in noncoding RNAs in disease.

miRNA could trigger the decay of lncRNAs. The abundance of numerous lncRNAs is controlled by miRNAs. The stability of lincRNA-p21 was regulated by the miRNA let-7b, whereas let-7b overexpression facilitated lincRNA-p21 degradation [96]. The recruitment of let-7 also caused the decreased stability of another lncRNA, HOTAIR, suggesting that the mechanism of lncRNA decay-enhanced miRNA interactions may be widespread. In some cases, lncRNAs serve as the precursor genes of the miRNAs with which they are co-transcribed. Studies have shown that H19 produces miR-675-3p and miR-675-5p in order to promote skeletal muscle differentiation and regeneration [97].

In addition, lncRNAs could indirectly regulate target genes by acting as decoys that sequester regulatory factors. Other studies have indicated that lncRNAs could regulate miRNAs at the transcriptional level. For example, lncRNA-MEG3 suppressed miR-21 expression in ECs and promoted the expression of RhoB and PTEN, subsequently modulating cell proliferation [81].

In addition, some lncRNAs, namely ceRNAs, act as sponges to prevent miRNAs from binding to their targets. lncRNA-RNCR3 functions as a ceRNA to regulate Kruppel-like factor 2 (KLF2) levels by sponging miR-185-5p in endothelial cells. lnc-MKI67IP-3 acts as a ceRNA for let-7e, thereby suppressing the proinflammatory effects of let-7e, and let-7e decreases lnc-MKI67IP-3 expression, thereby forming a positive feedback loop that aggravates inflammation. Whereas the mechanistic role of lncRNAs or miRNAs has been reviewed extensively above, the potential of these transcripts as novel biomarkers in CAD appears intriguing, especially since altered expression levels might represent different stages of CAD.

## Conclusion

The noncoding RNAs, especially miRNAs and lncRNAs, have been identified as key regulators in processes related to CAD caused by atherosclerotic lesions, and they play critical roles in several aspects of these processes, such as lipid metabolism, endothelial cell dysfunction, VSMC phenotype, cholesterol transport, foam cell formation, and vascular inflammation. Several miRNAs and lncRNAs have potential as biomarkers and as therapeutic targets for the diverse pathological changes involved in the CAD process. Further investigation of the functions of miRNAs and lncRNAs in CAD and elucidation of the responsible mechanisms needs to be performed.
